# Bioinformatic and experimental characterization of *SEN1998*: a conserved gene carried by the *Enterobacteriaceae*-associated ROD21-like family of genomic islands

**DOI:** 10.1038/s41598-022-06183-x

**Published:** 2022-02-14

**Authors:** Alejandro Piña-Iturbe, Guillermo Hoppe-Elsholz, Paulina A. Fernández, Carlos A. Santiviago, Pablo A. González, Susan M. Bueno

**Affiliations:** 1 Millennium Institute on Immunology and Immunotherapy, Facultad de Ciencias Biológicas, Departamento de Genética Molecular y Microbiología, Pontificia Universidad Católica de Chile, Santiago, Chile; 2 Laboratorio de Microbiología, Departamento de Bioquímica y Biología Molecular, Facultad de Ciencias Químicas y Farmacéuticas, Universidad de Chile, Santiago, Chile

**Keywords:** Bacterial genetics, DNA-binding proteins, Gene expression

## Abstract

Genomic islands (GIs) are horizontally transferred elements that shape bacterial genomes and contributes to the adaptation to different environments. Some GIs encode an integrase and a recombination directionality factor (RDF), which are the molecular GI-encoded machinery that promotes the island excision from the chromosome, the first step for the spread of GIs by horizontal transfer. Although less studied, this process can also play a role in the virulence of bacterial pathogens. While the excision of GIs is thought to be similar to that observed in bacteriophages, this mechanism has been only studied in a few families of islands. Here, we aimed to gain a better understanding of the factors involved in the excision of ROD21 a pathogenicity island of the food-borne pathogen *Salmonella enterica* serovar Enteritidis and the most studied member of the recently described *Enterobacteriaceae*-associated ROD21-like family of GIs. Using bioinformatic and experimental approaches, we characterized the conserved gene *SEN1998*, showing that it encodes a protein with the features of an RDF that binds to the regulatory regions involved in the excision of ROD21. While deletion or overexpression of *SEN1998* did not alter the expression of the integrase-encoding gene *SEN1970*, a slight but significant trend was observed in the excision of the island. Surprisingly, we found that the expression of both genes, *SEN1998* and *SEN1970*, were negatively correlated to the excision of ROD21 which showed a growth phase-dependent pattern. Our findings contribute to the growing body of knowledge regarding the excision of GIs, providing insights about ROD21 and the recently described EARL family of genomic islands.

## Introduction

Genomic islands (GIs) are genetic elements acquired by bacteria through horizontal gene transfer that are found integrated at the 5’ or 3’-end of different genes, including tRNA and tmRNA genes, and usually have sequence signatures (e.g. GC content, codon usage bias, dinucleotide frequency) that are distinct from those of the host genome^1–4^. GIs carry genes that encode advantageous functions for their bacterial hosts such as those related to metabolism of new substrates, antibiotic resistance or pathogenicity^5–7^, thus contributing to the survival and adaptation of bacteria in different environments. In addition to advantageous functions, many GIs also encode the molecular machinery responsible for their excision as circular extrachromosomal elements. Then, the circular form of GIs can be reintegrated or be the substrate for a new event of horizontal transfer by conjugation or high-frequency transduction in hijacked bacteriophage capsids^8,9^. Interestingly, besides its role in horizontal transfer, the excision of GIs can play a role in the virulence of plant and animal pathogens as found in *Pseudomonas syringae* pathovar *phaseolicola* and *Salmonella enterica* serovar Enteritidis, in which changes in the expression of genes inside the islands have been observed in response to the integrated/excised state of the GI^10,11^.

The excision/integration mechanism of GIs is thought to be similar to the mechanism described for lysogenic bacteriophages since the excision/integration module of both genetic elements encode similar genes and regulatory regions. The two attachment regions that flank the integrated Lambda phage, *attL* and *attR*, are the result of the recombination between the *attB* insertion site in the bacterial chromosome and the *attP* region in the circular phage DNA; *attL* and *attR* are populated by several binding sites for the different proteins involved in the assembly of the excisive intasome, the high-order nucleoprotein complex required for the excision of the prophage^12^. Lambda- and host-encoded proteins bind the *attL* and *attR* regions to bend the DNA and promote the intasome assembly, allowing the phage-encoded tyrosine recombinase, the integrase, to catalyze a site-specific recombination between the direct repeated sequences (DRSs) located at the end of each attachment region^13,14^. This recombination regenerates the insertion site *attB* and the circular form of the Lambda DNA and its *attP* region. While the Lambda integrase participates in the integration and excision reactions, the phage-encoded recombination directionality factor (RDF) Xis is key for excision, since it promotes the assembly of the excisive intasome, inhibiting integration^15,16^. As in prophages, GIs are also flanked by DRSs and encode integrases that catalyze the excision and integration of the island, and several GIs also encode one or more RDFs which participate in the excision process. While excision is being studied for some important families of GIs such as the SXT/R391^17,18^, the MGIs^19,20^, the SGI^21^ and more recently the tripartite symbiosis islands of *Mesorhizobium*^22,23^, there are several other GI families in which the particularities of their excision/integration process and its role in bacterial pathogenesis remain unknown.

The Region of Difference (ROD) 21 is one of the several GIs harbored by *Salmonella* ser. Enteritidis^24^. This island carries several genes (*SEN1970* to *SEN1999*) that encode proteins related to virulence, conjugal transfer, integration/excision, and others of unknown function^8,25–27^. Interestingly, the excision of ROD21 plays an important role in the invasion of the gastrointestinal epithelium and the subsequent colonization of deep organs in mice by *Salmonella* ser. Enteritidis, since mutant strains unable to excise ROD21 had a significantly reduced capacity to colonize the spleen, liver and gallbladder compared to the wild-type strain in which ROD21 is excised normally^11,25^. This phenomenon was hypothesized to be due to a regulatory mechanism in which gene expression within ROD21 is modulated by the supercoiling of the island, which may differ between its integrated and excised states, as observed for the PPHGI-1 island of *Pseudomonas syringae* pv. *phaseolicola*^11,28^. Hence it was hypothesized that the ROD21 excision may be part of a fine regulatory mechanism which assures that expression of the ROD21-encoded genes occurs at specific phases of the *Salmonella* ser. Enteritidis infective cycle^29^.

In a previous work, we identified the pathogenicity island ROD21 as a member of the *Enterobacteriaceae*-associated ROD21-like (EARL) GIs, a family of excisable islands distributed among different bacterial families of the order Enterobacterales, which share a set of conserved genes likely involved in the excision/integration and transfer mechanisms, and their regulation^7,30^. To gain insights regarding the genes involved in the excision of ROD21, we centered our attention on *SEN1998*, an ubiquitous gene among the EARL GIs, whose overexpression was previously found to increase the excision of ROD21^7,11^, although further characterization of its role was pending. Here, we describe the main features of *SEN1998* and its protein product through bioinformatic and experimental analyses.

## Results and discussion

### The gene *SEN1998* of ROD21 encodes a recombination directionality factor which is conserved among the EARL family of GIs

**Figure 1:**
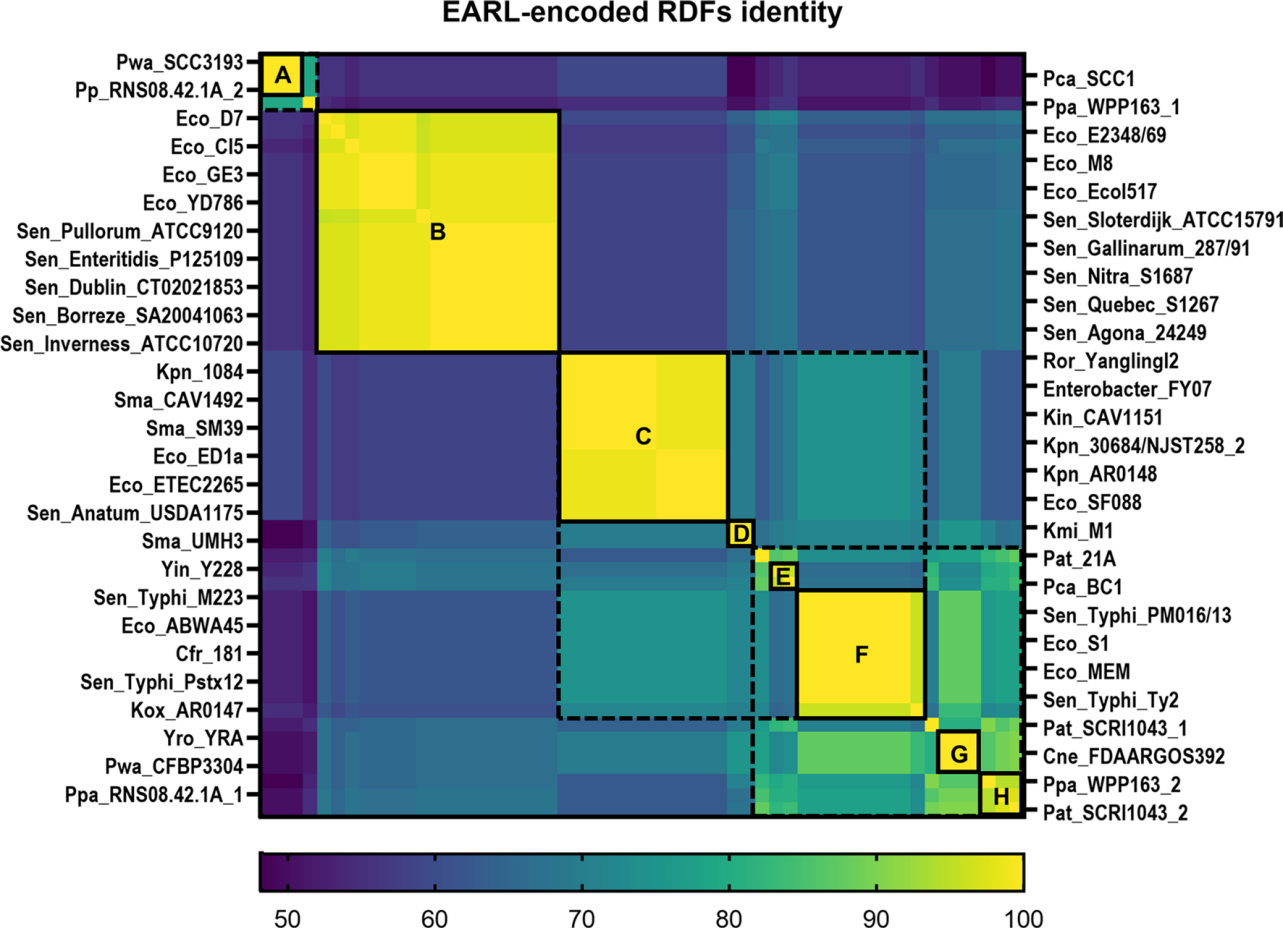
The SEN1998 homologs encoded by the EARL GIs share high level of amino acid identity. Heatmap representation of the identity matrix resulting from the multiple sequence alignment of the amino acid sequences of SEN1998 and the homolog proteins encoded by the EARL family of GIs. The color represents identities in the range from 48.15% to 100.00%. Clusters of proteins with identities ≥ 90% and ≥ 70% are delimited by solid and dashed lines, respectively. The name of the bacterial strains harboring the EARL GIs are indicated at each side of the heatmap.

**Figure 2:**
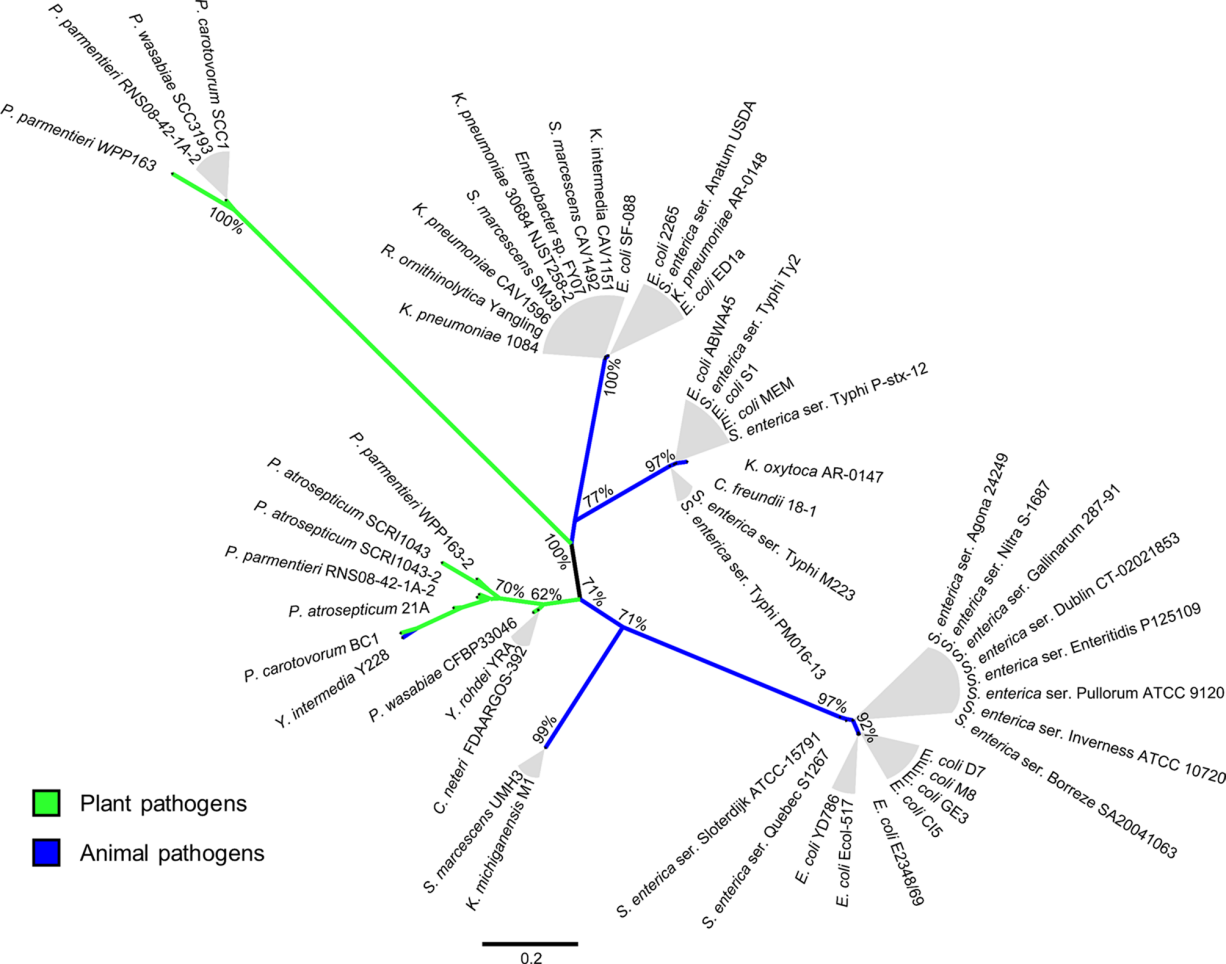
*SEN1998* and its homologs encoded by plant and animal pathogenic bacteria cluster together according to phylogeny. Maximum likelihood unrooted tree of SEN1998 and its homologs encoded by the EARL GIs. Green and blue branches indicate proteins encoded by islands integrated in the chromosomes of plant and animal pathogenic bacteria, respectively. The node support calculated from 5,000 bootstraps is indicated for the basal nodes only.

Since homologs of *SEN1998* were encoded by almost every identified member of the EARL GI family^7^, we decided to start our analyses comparing the amino acid sequences of their predicted proteins (60 to 72 amino acids) corresponding to 54 homologs, including SEN1998. A heatmap depicting the amino acid identity matrix of SEN1998 and its homologs shows that these proteins had identities that ranged from 48.15 to 100% (Fig. 1), indicating a relatively high level of conservation of these proteins among different GIs, specially within specific clusters of high identity that were denominated with letters from A to H (≥ 90%; Fig. 1A–H). Clusters A, B and H, comprised the proteins encoded by the GIs harbored mainly by strains of *Pectobacterium* spp., *Escherichia coli* and *Salmonella enterica*. However, the remaining clusters C to G comprised the proteins found in a higher diversity of bacterial species, such as *Klebsiella pneumoniae*, *Serratia marcescens*, *Salmonella* ser. Typhi, *E. coli*, *Citrobacter freundii* and *Enterobacter* sp., among others. The proteins from clusters C to F and E to H could also be respectively grouped in two ampler clusters of ≥ 70% identity, suggesting a closer relationship between those proteins (Fig. 1C–H, dashed lines). The same clustering described above was also observed in the phylogenetic tree constructed from the codon-aligned nucleotide sequences of the genes encoding these proteins (Fig. 2), which largely resembled the phylogenetic relationships previously reported for the integrase-encoding genes^7^, including the clustering of *SEN1998* and its homologs in groups encoded by plant-associated and animal-associated bacteria (Fig. 2; green and blue branches). Since integrases catalyze excision, the first step for the transfer of GIs, the agreement between their phylogeny and that observed for *SEN1998* and its homolog genes suggests an important role of the latter on dissemination and core functionality of the EARL GIs.

**Figure 3:**
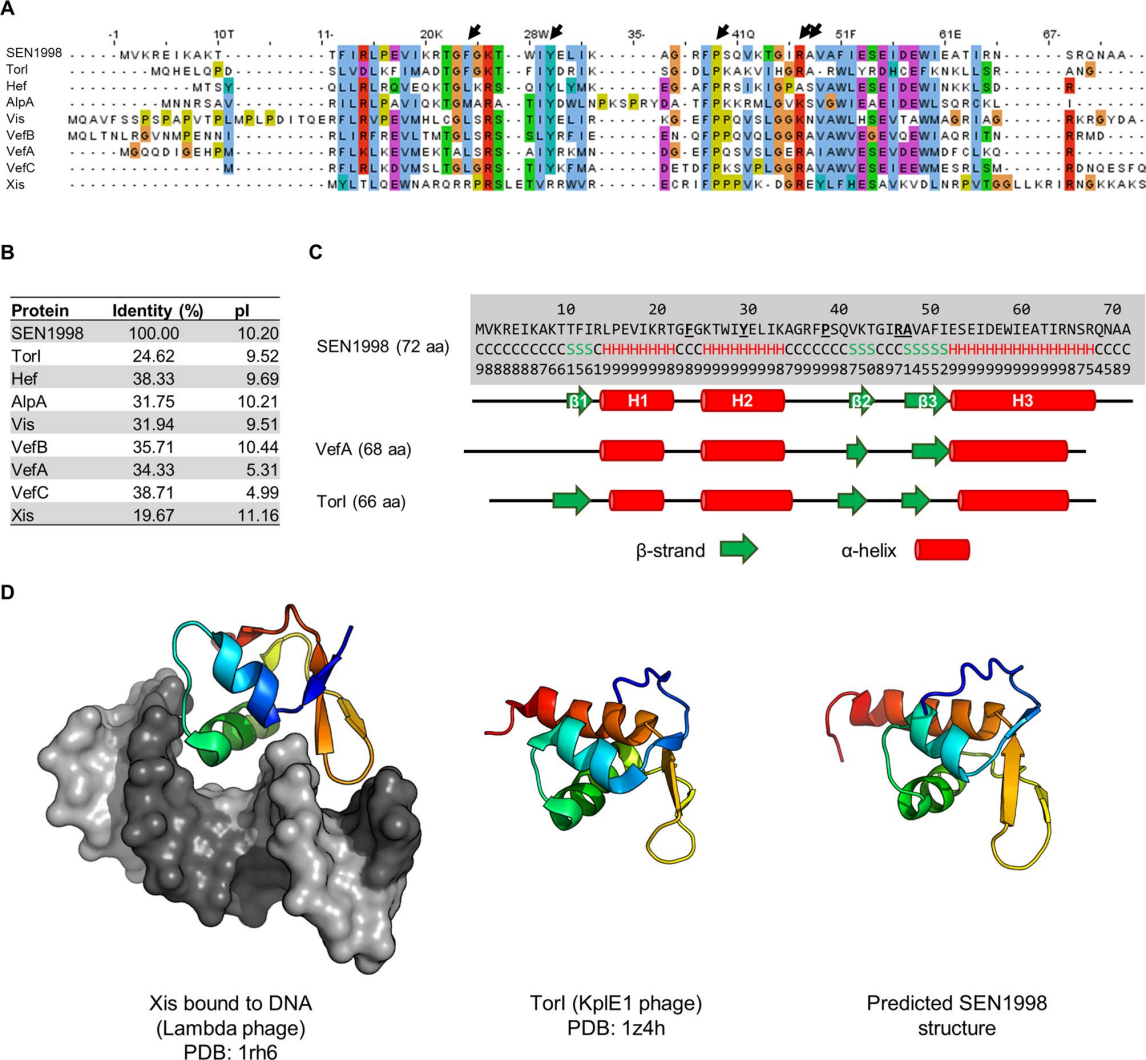
The gene *SEN1998* of ROD21 encodes a putative recombination directionality factor. (**A**) Multiple sequence alignment of the amino acid sequence of SEN1998 and characterized RDFs from phage (TorI, AlpA, Vis and Xis) and GI (Hef, VefA, VefB and VefC) origin. Conserved residues are colored according to the ClustalX coloring scheme. The black arrows indicate the conserved residues that has been recognized as important for the RDF function of TorI. (**B**) Percent identity of the aligned proteins regarding SEN1998, and isoelectric point. (**C**) Comparison of the secondary structures of SEN1998 (predicted), VefA (predicted)^33^, and TorI (experimentally determined)^35^. (**D**) Tertiary structures of the RDFs: Xis bound to DNA, (experimentally determined)^34^; TorI (experimentally determined)^35^ and SEN1998 (predicted).

To gain insights on the role of *SEN1998* and its predicted product, we compared SEN1998 (72 aa) with nine characterized RDFs and transcriptional regulators from phage and GI origin by multiple sequence alignment. The alignment showed the presence of conserved amino acid residues along the protein sequence from positions 11 to 60, including the conserved Phe-24, Tyr-30, Pro-39 and Arg-47, which are important for the RDF activity of TorI during the excision of the KplE1 prophage^31^ (Fig. 3A). The resulting identities relative to SEN1998 ranged from 19.67 to 38.70%, with the higher values belonging to the RDFs of the *Vibrio cholerae* Seventh Pandemic Island II (VefA) and the *Yersinia pestis* High-pathogenicity Island (Hef) (Fig. 3B). Conversely, SEN1998 shared a lower identity with TorI and Xis, the RDFs of the KplE1 prophage and the Lambda phage, respectively. While the isoelectric point of RDFs can be variable, the majority are basic proteins, a feature that may favor their binding capacity to the negatively charged DNA sugar-phosphate backbone^32^. In agreement with this feature, the calculated isoelectric point of SEN1998 resulted to be highly basic (pI = 10.20; Fig. 3B).

To further characterize SEN1998, the secondary and tertiary structures were predicted. The secondary structure of SEN1998 consisted of a β-strand (β1: Thr-11 to Ile-13) followed by two α-helices (H1: Leu-15 to Thr-22; H2: Lys-26 to Lys-34), two more β-strands (β2: Val-42 to Thr-44; β3: Ala-48 to Ile-52) and finally an α-helix longer than the previous ones (H3: Glu-53 to Arg-68) (Fig. 3C). This arrangement of secondary structures is conserved among RDFs, such as TorI^31^ (PDB: 1z4h) and the Vef proteins^33^ (predicted), where the helix H2 and the turn between strands β2 and β3 forms a winged-helix DNA-binding domain that enters the major and minor grooves, respectively, as observed in the structure of Xis^34^ (PDB: 1rh6) and TorI^35^ (Fig. 3D). The predicted tertiary structure of SEN1998 is highly similar to that of TorI and Xis, and has the winged-helix motif, suggesting the DNA binding capacity of SEN1998. Together, the sequence analysis strongly suggests that SEN1998 is an RDF.

### The SEN1998 protein binds the two attachment regions of ROD21 with different affinities

**Figure 4:**
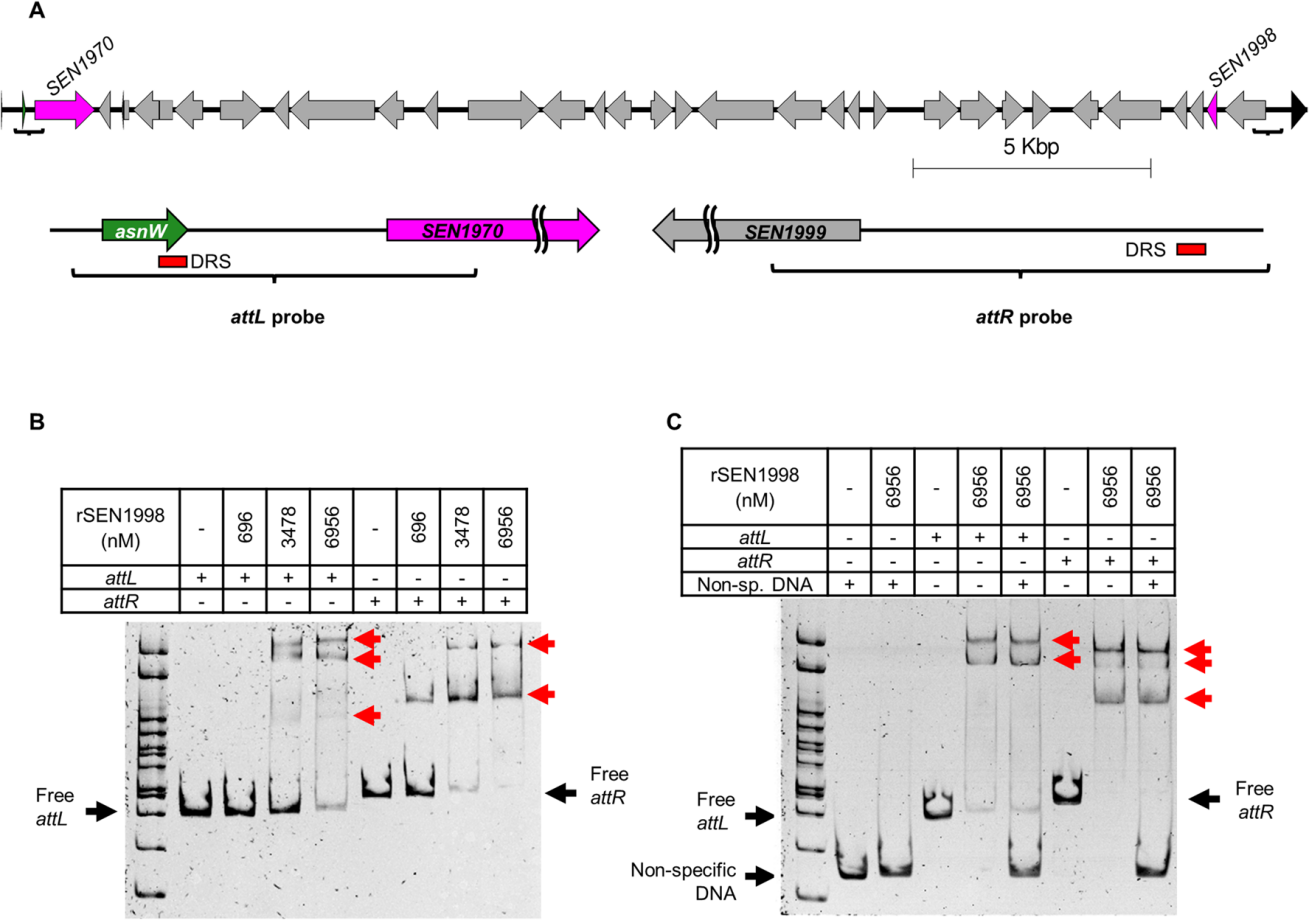
The SEN1998 protein binds the two attachment regions of ROD21 with specificity. (**A**) Schematic representation of the target DNAs encompassing the left and right attachment regions of ROD21 used for the EMSAs. (**B**, **C**) EMSAs showing the binding of SEN1998 to both *att* regions, forming different complexes. The black and red arrows indicate the free and complexed target DNA. The multiple cloning site of plasmid pGEM-T Easy was used as a non-specific competitor DNA in **C**.

To further characterize the product of *SEN1998*, we cloned its coding sequence in a pET-15b vector and purified the 6x-His-tagged recombinant protein (Supplemental Fig. S1). Then, we assessed the DNA-binding capacity of SEN1998 through electrophoretic mobility shift assays (EMSAs). Since most of the studied RDFs bind several sites at one of the attachment regions of their prophage or GI to promote excision^33,36^, we decided to use the left and right attachment regions of ROD21 as the target DNA. Interestingly, we found that SEN1998 can bind the two attachment regions of ROD21, forming different complexes with different mobilities (Fig. 4; not cropped gels in Supplemental Fig. S2). This suggests the presence of more than one binding site in each *att* region and, therefore, the participation of more than one SEN1998 protein in the formation of the excisive intasome of ROD21. This finding is in agreement with observations made for prophages, whose RDFs bind at multiple sites to assist the formation of the DNA curvature necessary for intasome assembly^37^. The SEN1998-*att* complexes became visible at 3.5 µM for *attL* and 0.7 µM for *attR*, suggesting a higher binding affinity of SEN1998 for the *attR* region (Fig. 4B). When a non-specific competitor DNA was included in each binding assay, no other complexes were detected besides those formed between SEN1998 and the *att* regions in the absence of the competitor, indicating that the binding of SEN1998 is specific to the attachment regions of ROD21 (Fig. 4C). Our results also suggest that the binding sites of SEN1998 are located at the distal part of the *att* regions (i.e., closer to the DRSs), since binding of SEN1998 to shorter target DNAs was observed only for those that span the island boundaries (Supplemental Figure S3).

### The excision of ROD21 and the expression of *SEN1998* and *SEN1970* are modulated by the growth phase

To further explore the role of *SEN1998* in the excision of ROD21, we evaluated the excision pattern of the island along the growth curve of the wild-type strain and quantified the expression of *SEN1998*.

**Figure 5:**
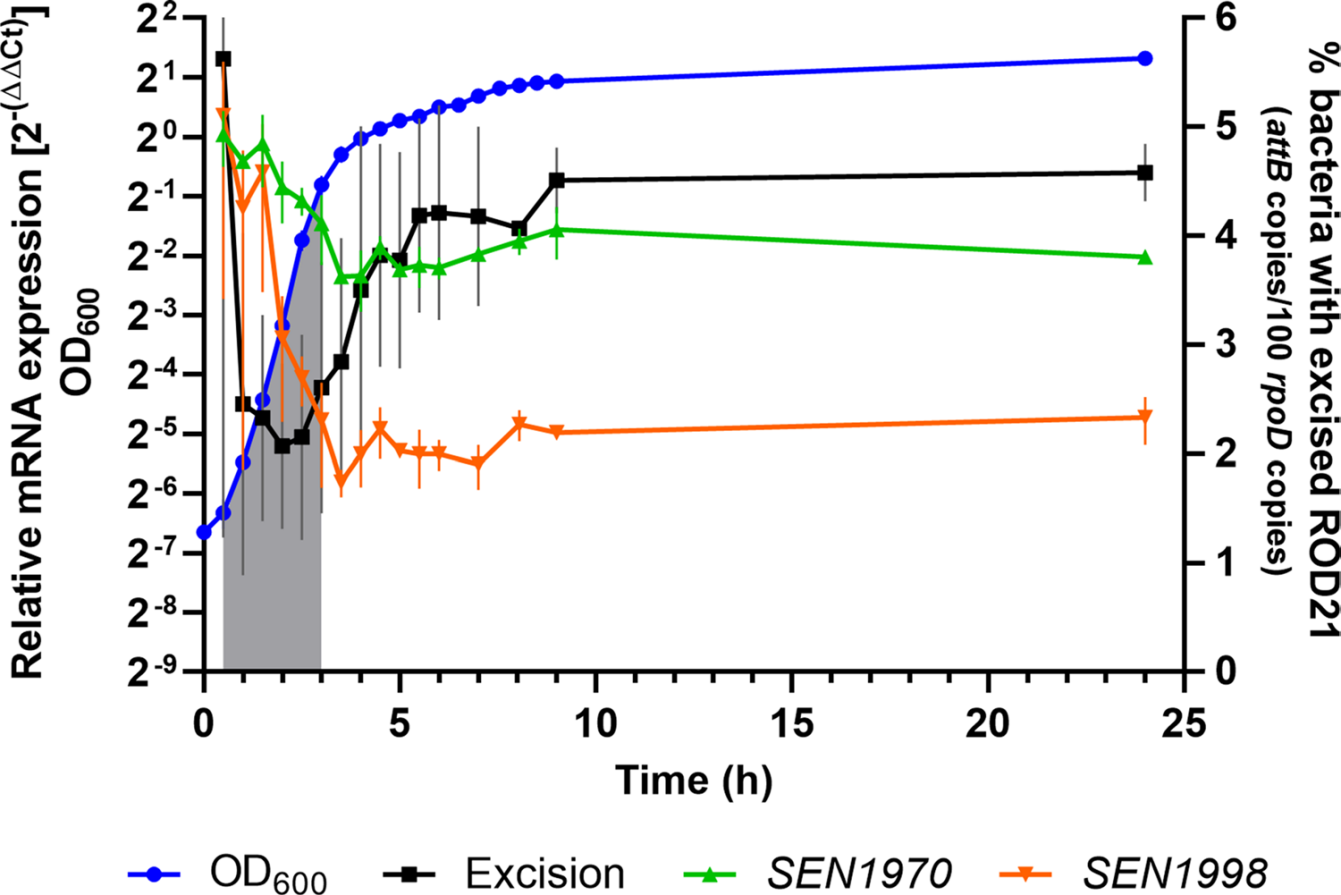
The excision of ROD21 increases during the stationary phase of growth as the expression of *SEN1970* and *SEN1998* decreases. ROD21 excision, and *SEN1970* and *SEN1998* expression patterns during the growth of *Salmonella* ser. Enteritidis P125109 in LB at 37 °C with shaking. The gray area represents the logarithmic phase of growth. Values are the mean ± SD of two independent experiments.

The excision of GIs is usually expressed as the ratio between the copy number of the empty *attB* site and a single-copy gene (e.g. *attB*/*rpoD*), a value that varies between 0 (all *attB* sites occupied; *attB* = 0) and 1 (all *attB* sites empty; *attB* = *rpoD*). Since this ratio is an estimation of the population fraction with an excised GI, we decided to represent it as a percentage of the population (*attB*/100 *rpoD*) to provide a straightforward representation of the excision level within the bacterial population. We found that the excision reaches its lower values during the logarithmic phase (between 2.1 and 2.6%) and then increases in the transition to stationary phase to stabilize in a maximum value during the stationary phase (around 4.5%) (Fig. 5; black line). While not all GIs have an excision pattern like the observed for ROD21 (see for example ref.^2^), the excision increase upon entrance to stationary phase has been observed for the ICE*Ml*Sym^R7A^ symbiosis island of *Mesorhizobium loti* R7A^38^ and the ICE*St1* and ICE*St3* islands of *Streptococcus thermophilus* CNRZ368 and CNRZ385^39^. As *rpoD* is located at 0.8 Mbp from the *oriC* and the integration site of ROD21 is approximately at 1.9 Mbp from the origin, it is possible that gene dosage effects, resulting from active chromosome replication, could interfere with accurate estimations of the excision level in the bacterial population by increasing the *rpoD* copy-number in a determined sample, thus reducing the quantified excision by one half or more (Excision = *attB*/100 *rpoD*). However, when the *attB* and *rpoD* copy-numbers from the same sample are plotted as pairs, it becomes evident that there are not high numbers of *rpoD* associated to low *attB* numbers. This is, the higher the *rpoD* copies, the higher the *attB* copies (Supplemental Fig. S4), as expected for variations due to the DNA concentration in the sample, since dilutions at 40 to 60 ng/µL (about 8 × 10^6^ to 1.2 × 10^7^ *rpoD* copies) were used in every copy-number determination. Therefore, the potential effect of the gene dosage as a result of the location of *rpoD* relative to the *oriC* is negligible.

The increased excision of ICE*St1* and ICE*St3* can be explained by the higher expression levels of the genes encoding their corresponding RDFs and also their integrases^39^. Unexpectedly, when we quantified the expression of *SEN1998*, a marked decrease in its expression was found (Fig. 5; orange lines), showing a reduction of about 70 times their initial level at 0.5 h. We also assessed the expression levels of the integrase-encoding gene *SEN1970* in the same samples, finding a decrease in its expression as well (about 5 times; Fig. 5, green lines). The decrease in the expression of both genes occurred during the log phase while the excision of ROD21 remained nearly constant; then, the expression remained with little variation along the transition and stationary phases (Fig. 5). In other study, it was found that the supercoiling of the excised form of the PPHGI-1 island of *Pseudomonas syringae* pv. *phaseolicola* was involved in the repression of the genes carried by this GI^10^. We have previously observed expression changes in the genes contained within ROD21 in response to an impaired excision capacity^11^, which suggest a role of the excised/integrated state of ROD21 in the modulation of gene expression. Nevertheless, the downregulation of *SEN1970* and *SEN1998* observed in our experiment may not be related to the excised/integrated state of ROD21 since the decrease in the expression levels occurred when the excision level was nearly constant (Fig. 5). While these results suggest an unknown regulatory mechanism that represses the expression of *SEN1970* and *SEN1998* during the phase of active growth, it is not clear how these changes affect the excision of ROD21 which starts to increase as the expression of both genes stabilizes around its minimum.

### The excision of ROD21 significantly differs in strains lacking and overexpressing *SEN1998*

Previous studies have shown that the overexpression of *SEN1998* increases the excision level of ROD21 within the bacterial population^8,11^; however, our previous experiment suggests that this is not the case and that low expression levels correlates with a higher excision. To further assess how *SEN1998* contributes to the excision of ROD21, we constructed two derivatives of *Salmonella* ser. Enteritidis strain P125109: one mutant strain lacking the coding sequence of SEN1998 (Δ*SEN1998*::*frt*) (Supplemental Fig. S5) and the isogenic complemented strain that harbors the plasmid pTrc-*SEN1998* for IPTG-inducible expression.

**Figure 6:**
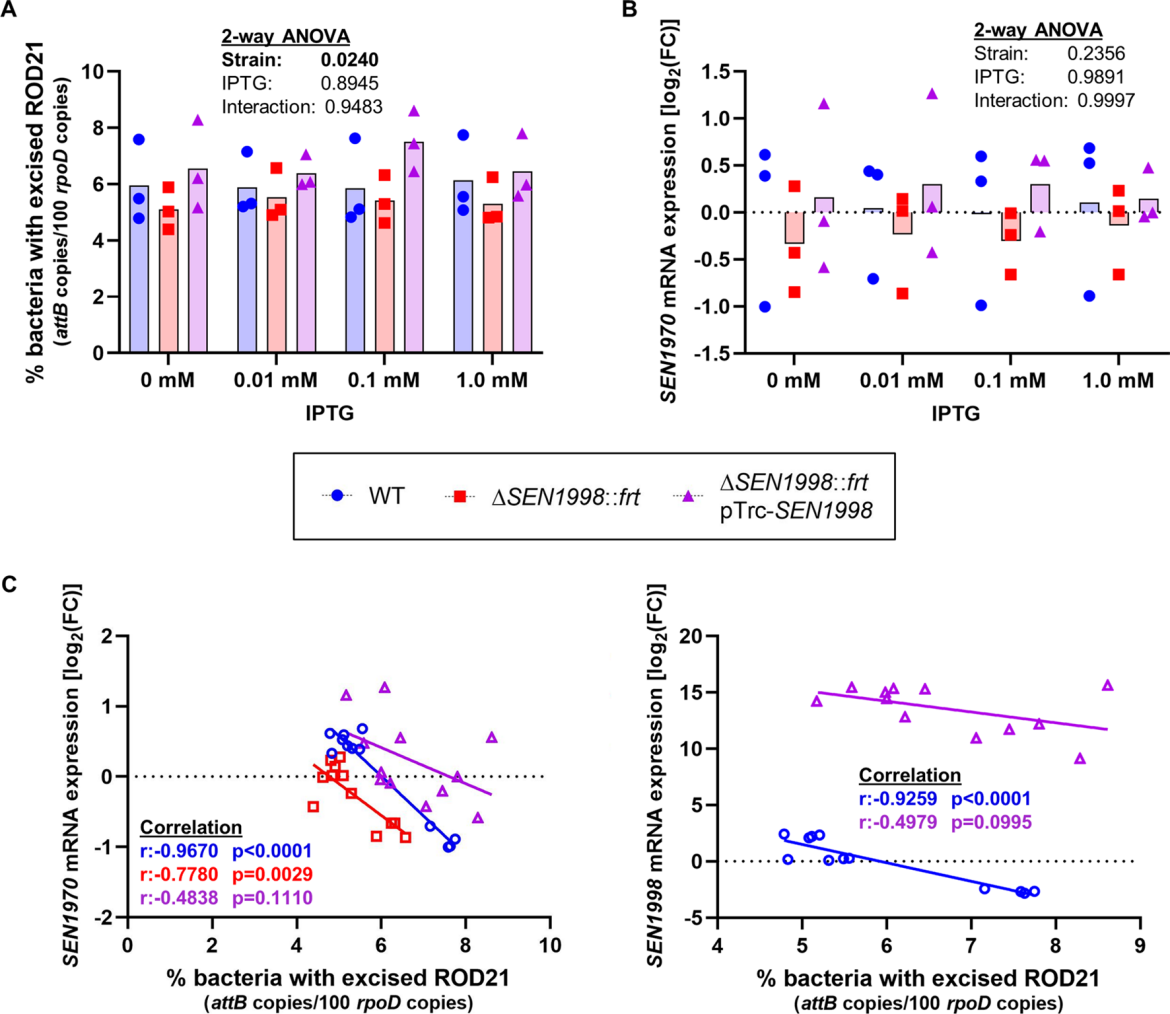
Effect of the absence and overexpression of *SEN1998* and correlation of the excision with the *SEN1970* and *SEN1998* expression. (**A**) Excision levels of ROD21, represented as the percentage of the bacterial population with an empty *attB* site, in *Salmonella* ser. Enteritidis P125109, Δ*SEN1998*::*frt* and Δ*SEN1998*::*frt* pTrc-*SEN1998*, grown in the presence of different concentrations of IPTG. (**B**) mRNA levels of the integrase-encoding gene *SEN1970* in the three strains, relative to the levels in P125109. Bars in A and B represent the mean of three biological replicates (individual points), at a specific IPTG concentration. (**C**) Scatter plot of the relative *SEN1970* (left panel) and *SEN1998* (right panel) mRNA levels versus the excision level in the same sample for the three *Salmonella* strains. The Pearson correlation coefficient and p-value are shown for each strain.

The excision level in the wild-type strain *Salmonella* ser. Enteritidis P125109 showed a high variability between independent experiments, ranging from 4.8% to 7.7% of the bacterial population (Fig. 6A; blue symbols). While this result is in accordance with previous observations in *Salmonella* ser. Enteritidis^7,11,25^, it is to be noted that the excision frequency of ROD21 in LB is considerably high when compared to other GIs whose excision frequencies in uninduced conditions are usually very low (< 0.1%)^2,17,23,38,40^. When we deleted the *SEN1998* coding sequence, the excision decreased slightly (4.4% to 6.6%) and, although the differences were not statistically significant when compared to the wild-type strain, this trend was observed in 11 out of 12 different measures (Fig. 6A, red symbols; Supplemental Fig. S6). When the deletion of *SEN1998* was complemented from a plasmid, the excision levels increased again, ranging from 5.2 to 8.6%, and equaled or surpassed the excision level of the wild-type strain (Fig. 6A, purple symbols). The expression of *SEN1998* from the pTrc-*SEN1998* plasmid was leaky, and reached very high levels, from 1396 to 37,666 times the wild-type level in uninduced and 1.0 mM IPTG conditions, respectively (Supplemental Fig. S7). It is possible that the high expression levels of *SEN1998* could interfere with the synthesis of functional proteins, which may explain why, in other studies, the overexpression of *SEN1998* from the pBAD-*SEN1998* plasmid resulted in a higher excision level in the wild-type strain (≈20%)^11^. However, when we overexpressed *SEN1998* from IPTG- and L-arabinose-inducible promoters in a low-copy number plasmid (pAPI-*SEN1998*) and from pBAD-*SEN1998* trying to reproduce previous observations, the excision levels remained similar to those of the wild-type strain (Supplemental Fig. S8). Nevertheless, the observed pattern in Fig. 6A, of lower excision levels in the mutant strain and the recovery in the complemented strain, supports the involvement of *SEN1998* in the excision of ROD21. Indeed, the two-way ANOVA indicated that statistically significant differences existed between the means regarding the “strain” factor (p = 0.0240). The Tukey post-hoc test comparing the main effect of the “strain” factor revealed statistically significant differences (p = 0.0462) between the excision levels of the mutant Δ*SEN1998*::*frt* and its isogenic complemented strain.

As the excision reduction that results from deleting *SEN1998* is very low, the contribution of *SEN1998* to the excision of ROD21 seems to be non-essential. It has been previously observed for other GIs that the deletion of the RDF-encoding gene usually produces a significant to moderate decrease of the excision frequency and, in many cases, the absence of the RDF did not abolish the excision capacity, indicating that these proteins are not essential for the excision of their cognate GIs^17,23,33,38,41,42^. For example, the island IE3 carried by *E. coli* E2348/69 is an EARL GI that carries the CDS of a SEN1998 homolog with a point mutation in the start codon (ATG > ACG) which disrupted the CDS. We have previously demonstrated that IE3 is excision-proficient^7^. It is also possible that a different RDF encoded outside ROD21 could participate in its excision, as was observed for the VPI-2 genomic island of pandemic *Vibrio cholerae* strain N16961^33^. However, a tBLASTn search of *SEN1998* homologs in the genome of *Salmonella* ser. Enteritidis P125109 (GenBank accession NC_011294.1) did not retrieve any homolog sequence (data not shown).

### The expression of *SEN1998 *and the integrase-encoding gene *SEN1970* are negatively correlated with the excision of ROD21

It is not clear why a gene encoding an RDF-like protein not essential for excision is highly conserved among all the GIs belonging to the EARL family^7^. Perhaps, SEN1998 and their homologs could play additional roles important for the excision or transfer of their cognate GIs. For example, in the KplE1 prophage and the VPI-1 and VPI-2 GIs, it has been observed that their RDFs can negatively modulate the expression of the integrase as a result of the overlap between the *att* region, bound by the RDF, and the integrase promoter^33,43^. Therefore, we aimed to assess whether SEN1998 can modulate the expression of *SEN1970*, since the *SEN1970* promoter is expected to be in the *attL* region bound by SEN1998 (Fig. 4). Quantification of the *SEN1970* expression in the Δ*SEN1998*::*frt* and complemented strains revealed slight variations that were not statistically different from the expression level in the wild-type strain (Fig. 6B). Interestingly, the same trends observed for excision were also present in the expression of the integrase-encoding gene: lower levels of *SEN1970* expression in the strain lacking *SEN1998* (Fig. 6B, red symbols) and a higher expression in the complemented strain (Fig. 6B, purple symbols). Although these results may suggest a role for SEN1998 in the expression of the integrase SEN1970, it would not be as a negative regulator but as an activator. Additionally, the observed changes in expression can be a consequence of the supercoiling of ROD21 after excision and not a result of the expression of *SEN1998*. Interestingly, a closer examination of the expression and excision values revealed a negative correlation between the expression of *SEN1970* and *SEN1998*, and the excision of ROD21 (Fig. 5C), which is: the higher the excision, the lower the expression of both genes in the same sample. This finding supports our observation of a decrease in the *SEN1970* and *SEN1998* mRNA levels along the growth curve (Fig. 5), and is in agreement with a previous study in which the increase of the ROD21 excision correlated with a reduction in the expression of *SEN1970* when bacteria were grown in the presence of hydrogen peroxide^25^. Experiments using translational reporters would be useful to assess how the SEN1998 protein levels correlate with the *SEN1998* mRNA and with the excision of ROD21.

## Concluding remarks

In this study, we aimed to gain a better understanding of the factors involved in the excision of the pathogenicity island ROD21, focusing on the gene *SEN1998*. While bioinformatic and DNA-binding analyses shown that *SEN1998* encodes a protein that shares all the main features of an RDF that specifically binds the attachment regions of ROD21, the experimental assessment of its role in the excision of the island revealed that *SEN1998* is not essential under the conditions tested. However, since *SEN1998* is highly conserved among the EARL GIs, it is probably playing important roles in the biology of these islands. As this family of GIs has been recently described, our understanding of their roles and biology is still incipient, and more research is needed. A case of interest would be the ICEKp258.2 island harbored by the globally-spread carbapenem-resistant *Klebsiella pneumoniae* ST258. The acquisition of this EARL member by the ancestral ST258 strain, in the last decade of the twentieth century^44,45^, has been suggested to be associated to its successful dissemination around the world^46^. Nevertheless, little is known about this excisable island. The excision of different families of GIs harbored by Gram-negative and Gram-positive bacteria is currently being studied by different groups, and a better understanding of the excision mechanism has proven useful to gain insights about the dissemination of virulence and resistance traits, the interaction of GIs with other horizontally acquired elements, and the evolution of bacterial pathogens^21,23,47–49^. Our findings contribute to the growing body of knowledge regarding the excision of GIs, providing insights on the recently described EARL family of genomic islands.

## Materials and methods

### Bacterial strains, plasmids and culture conditions

*Salmonella enterica* serovar Enteritidis P125109, *Escherichia coli* BL21(DE3), *E. coli* DH5α and DH5α λ*pir* were maintained in CRYOBANK tubes (VWR) at − 80 °C and cultured in LB broth with agitation or LB agar, at 37 °C or 30 °C, when needed. Plasmid pET15b-*SEN1998*, encoding a N-terminal 6xHis-tagged SEN1998 protein, was synthesized by Epoch Life Science and transformed by electroporation in *E. coli* BL21(DE3) for protein expression. Plasmids pKD3, pKD46 and pCP20, used for deletion of the *SEN1998* coding sequence, were maintained in *E. coli* DH5α λ*pir* (pKD3) and DH5α (pKD46; pCP20), and purified using the Wizard® Plus SV Minipreps DNA Purification System (Promega). When necessary, chloramphenicol (CHL) or ampicillin (AMP) were added to the culture media at 34 µg/mL and 100 µg/mL, respectively.

The mutant strain *Salmonella* ser. Enteritidis Δ*SEN1998*::*frt* was constructed using the Lambda Red system^50^ and the primers listed in the Supplemental Table S1. The chloramphenicol acetyl transferase (*cat*) cassette was amplified from the template plasmid pKD3 by PCR, using primers H1+P1_SEN1998_Fw and H2+P2_SEN1998_Rev, and purified using the Wizard SV PCR and Gel Clean-up System (Promega). *Salmonella* ser. Enteritidis P125109, harboring the pKD46 helper plasmid, was grown in LB broth at 30 °C until OD_600_ = 0.3. Then, 1 mM l-arabinose was used to induce expression of the Lambda Red proteins from pKD46 during 30 min. Electrocompetent cells were prepared and transformed with the purified *cat* cassette, and then incubated in approximately 2 mL LB broth at 30 °C during 2 h. After recovering, cells were pelleted, spread on LB-CHL agar, and incubated at 30 °C. After 72 h, CHL-resistant colonies were selected and grown in LB-CHL agar at 37 °C overnight. After incubation, one colony per clone was selected and subcultured again in LB-CHL agar at 37 °C. Liquid cultures in LB-CHL were prepared and used for DNA extraction and preparation of frozen stocks. Replacement of the *SEN1998* gene by the *cat* cassette was confirmed by PCR using specific primers for *SEN1998* (SEN1998_NcoI_Fw and SEN1998_HindIII_Rev), the *cat* cassette (H+P primers), primers external to the mutation site (SEN1997 3’ Fw and SEN1999 5’ Rev), and a combination of H1+P1_SEN1998_Fw and SEN1999 5’ Rev.

The mutation was transferred to a clean genetic background by generalized transduction using phage P22 HT105/1 *int*-201. Transducing P22 particles were obtained by infecting the *Salmonella* ser. Enteritidis Δ*SEN1998*::*cat* strain in 10 mL of LB-CHL broth with agitation at 37 °C, overnight. After adding 500 μL of chloroform, the culture was vigorously shaken, pelleted, and the clear supernatant was recovered, passed through a 0.22 µm syringe filter and stored at 4 °C. One hundred microliters of an overnight culture of wild-type *Salmonella* ser. Enteritidis P125109 were mixed with 900 µL of the transducing P22 suspension and incubated at 37 °C during 60 min. Then, 100 µL were plated on LB-CHL agar and incubated at 37 °C. CHL-resistant colonies were selected, and the presence of the *cat* cassette was confirmed by PCR as described above. The selected transductants were assessed for the presence of lysogens and pseudolysogens by plating on Evans blue agar and testing susceptibility to phage P22 H5. Finally, the lysogen/pseudolysogen-free transductant was electrotransformed with plasmid pCP20^51^ to eliminate the *cat* cassette by Flp-mediated excision, followed by growth of transformants at 42 °C for plasmid curing. The resulting *Salmonella* ser. Enteritidis Δ*SEN1998*::*frt* strain was verified by PCR, and CRYOBANK stocks were prepared and stored at − 80 °C.

### Bioinformatic analysis

Amino acid sequences of proteins SEN1998 (CAR33580.1), VefA (AAF94934.1), VefB (AAF94957.1), VefC (AAF93670.1), Hef (CAB46594.1), AlpA (NP_417113.1), Vis (NP_042041.1), Xis (NP_040610.1) and TorI (WP_001163428.1) were downloaded from GenBank. The isoelectric point of the nine proteins were calculated using the tool Compute pI/Mw from ExPASy (https://web.expasy.org/compute_pi/). The multiple sequence alignment was performed in MEGA X v.10.0.5^52^ using MUSCLE, and the resulting alignment was opened and colored in JalView v.2.10.3b^53^. Finally, prediction of the SEN1998 secondary and tertiary structures was carried out using the I-TASSER server^54^. Figures of the Xis bound to DNA (PDB: 1rh6), TorI (PDB: 1z4h) and predicted SEN1998 structures were obtained using PyMOL v.2.1 (https://pymol.org/2/).

The amino acid sequences of the SEN1998 homologs encoded by the EARL GIs were obtained from each GI^7^ and aligned using the web version of MUSCLE^55^. The corresponding pairwise identity matrix were downloaded and plotted as a heatmap in GraphPad Prism v.9.1.1.

An unrooted maximum likelihood tree was constructed for SEN1998 and its homologs. The codon-aligned nucleotide sequences were obtained using MUSCLE in MEGA X and subsequently uploaded to the IQ-Tree web server^56^ for prediction of the best-fitting nucleotide substitution model (TVM + F + G4) and construction of the phylogenetic tree. Nodes support was calculated from 5,000 bootstraps using the ultrafast approximation^57^. FigTree v1.4.3 (http://tree.bio.ed.ac.uk/software/figtree/) was used for tree visualization and color edition.

### Protein expression and purification

*Escherichia coli* BL21(DE3) pET15b-*SEN1998* was grown in 750 mL of LB-AMP broth at 37 °C and ≈220 rpm until OD_600_ = 0.8–1.0. The culture was cooled using ice, and the expression of the recombinant N-terminal-6xHis SEN1998 (rSEN1998) was induced with 1 mM IPTG during 20 h at room temperature (19–22 °C) and 220 rpm. Cells were harvested by centrifugation at 7200×*g* at 4 °C during 6 min and stored at − 30 °C until needed.

The cell pellet was resuspended in lysis buffer [20 mM phosphate buffer, 100 mM NaCl, 30 mM imidazole, 0.5 mM Tris(2-carboxyethyl)phosphine, 5% glycerol, ≥ 25 U/mL Benzonase, 2 mM MgCl_2_, 1X cOmplete™ Mini EDTA-free Protease Inhibitor Cocktail; pH 8.0] and incubated with 1 mg/mL lysozyme during 30 min at 37 °C and 200 rpm. After incubation, the suspension was cooled on ice and then was sonicated, avoiding overheating, with 16 pulses in groups of 4 pulses of 15 s each, using a Microson™ ultrasonic cell disruptor (Misonix) at level 10. The lysate was cleared by two steps of centrifugation at 4 °C (8228×*g*/6 min, and 15,557×*g* /45 min) and the supernatant was recovered and stored at 4 °C overnight.

rSEN1998 was purified by immobilized metal-ion affinity chromatography using HisTrap Fast Flow columns (Merck-Millipore). The column was equilibrated with 10 column volumes (CVs) of buffer A (20 mM phosphate buffer, 500 mM NaCl, 30 mM imidazole, 0.5 mM TCEP, 5% glycerol; pH 8.0), and the recovered supernatant was loaded. The column was washed with 30 CVs of buffer B (20 mM phosphate buffer, 500 mM NaCl, 150 mM imidazole, 0.5 mM TCEP, 5% glycerol; pH 8.0) and rSEN1998 was eluted with 10 CVs of Buffer C (20 mM phosphate buffer, 500 mM NaCl, 300 mM imidazole, 0.5 mM TCEP, 5% glycerol; pH 8.0). Eleven fractions of 1 mL were collected and those with the higher amount of rSEN1998 were pooled and dialyzed against Binding buffer (10 mM Tris–HCl, 150 mM KCl, 0.1 mM EDTA, 0.5 mM TCEP, 5% glycerol; pH 8.0). The protein was concentrated in an Amicon™ Ultra-4 centrifugal filter (MWCO = 3.5 kDa; Merck), and Binding buffer containing 50% glycerol was added to obtain a final concentration of 20% glycerol. Protein concentration was determined using the Pierce™ BCA Protein Assay (Merck) and the purity of rSEN1998 was estimated from a 15% polyacrylamide gel stained with Coomassie blue using ImageJ v.1.52a^58^. The protein was stored in frozen aliquots at − 80 °C.

### Electrophoretic mobility shift assays

The *Salmonella* ser. Enteritidis P125109 DNA containing the left and right attachment regions of ROD21 was amplified by PCR using the Phusion High-Fidelity DNA Polymerase (Thermo Scientific) with the HF Buffer and the primers attL_Fw/attL_Rev and attR_Fw/attR_Rev, respectively (Supplemental Table S1). The amplified regions correspond to the positions 2,061,048 to 2,061,443, and 2,087,253 to 2,087,698 in the *Salmonella* ser. Enteritidis P125109 chromosome (GenBank Accession Number NC_011294). The *attL* (360 bp) and *attR* (446 bp) PCR products were purified directly from the PCR tube using the Wizard® SV Gel and PCR Clean-Up System (Promega) and stored at − 30 °C in nuclease-free water. The multiple cloning site of plasmid pGEM-T Easy (261 bp) (Promega) was amplified, purified and used as a non-specific competitor DNA.

One hundred nanograms of the *attL* and/or the *attR* DNA were incubated with 0; 696; 3478; and 6956 nM rSEN1998 in Binding buffer (10 mM Tris–HCl, 150 mM KCl, 0.1 mM EDTA, 0.5 mM TCEP, 5% glycerol; pH 8.0) at room temperature during 60 min in a total volume of 20 µL. After incubation, the total volume was loaded in a native 6% polyacrylamide (37.5:1) gel prepared with 0.5X Tris–Borate-EDTA (TBE) buffer (Merck) that has been pre-run at 100 V during 60 min in 0.5X TBE. Samples were run at 70 V during 150 min and the gel was stained during 20 min in water with 5X GelRed® (Biotium) and visualized with UV light in a myECL™ Imager (Thermo Scientific). EMSAs with target DNAs corresponding to subregions of the *attL* and *attR* targets (*attL*-A, *attL*-B, *attR*-A and *attR*-B) were performed as described above, using purified PCR products obtained with primers pairs attL_Fw/attL-A_Rev, attL-B_Fw/attL_Rev, attR_Fw/attR-A_Rev and attR-B_Fw/attR_Rev (Supplemental Table S1).

### Assessment of the *SEN1970* and *SEN1998* expression and the ROD21 excision patterns along the growth curve

An overnight culture of *Salmonella* ser. Enteritidis P125109 was grown in 320 mL of LB broth, starting at OD_600_ = 0.01, during 24 h at 37 °C with shaking. Samples were taken each 30 min from time 0.5 h to 6.0 h, each hour from 7.0 h to 9.0 h, and then at time 24 h, and OD_600_ was measured. Samples were centrifuged at 6200×*g* during 6 min at 4 °C, the supernatant discarded, and pellets were stored at − 30 °C for DNA extraction and − 80 °C in 1 mL TRIzol for RNA extraction. Two independent biological replicates were carried out.

### Effect of *SEN1998* deletion on the excision of ROD21 and the expression of the integrase-coding gene *SEN1970*

Overnight cultures of *Salmonella* ser. Enteritidis P125109, Δ*SEN1998*::*frt* and Δ*SEN1998*::*frt* pTrc-*SEN1998* strains were grown in LB broth (37 °C, with shaking) until OD_600_ ≈ 0.6. Five mL of each culture were transferred to 50 mL conical tubes and IPTG was added to obtain final concentrations of 0.01, 0.1 or 1.0 mM. One tube remained without IPTG as a control. After 2 h incubation at the same conditions, 1.5 mL samples were taken and centrifuged at 6200 × g during 6 min at 4 °C. Supernatants were discarded, the pellets for DNA extraction were stored at − 30 °C and the pellets for RNA extraction were resuspended in 1 mL TRIzol (Invitrogen) and stored at − 80 °C. Three independent biological replicates, performed in duplicate, were carried out.

### Extraction and purification of genomic DNA and total RNA

Genomic DNA was purified from bacterial pellets stored at − 30 °C. Cells were resuspended in 550 µL of Tris–EDTA buffer (pH 8.0) followed by the addition of 5 µL of 5 mg/mL RNase A, 10 µL of 10 mg/mL proteinase K and 30 µL of 10% SDS. After incubation at 37 °C during 1 h, 600 µL (1 volume) of phenol:chloroform:isoamyl alcohol (PCI; Winkler) were added for DNA extraction. The tubes were vigorously shaken and centrifuged at 21,100×*g* during 15 min at 4 °C. The transparent aqueous phase (300 µL) was transferred to a new tube to repeat the extraction with PCI. This step was repeated again with 200 µL of the aqueous phase. After recovering 100 µL of the aqueous phase from the third extraction, 0.1 volumes of 3 M sodium acetate and 1 volume of isopropanol were added, followed by incubation at − 20 °C during 30 min for precipitation of the DNA. The tubes were centrifuged at 21,100×*g* during 30 min at 4 °C and the DNA pellet was washed with 0.5 mL of 75% ethanol by inverting the tube five times. After centrifugation at 7500×*g* during 5 min at 4 °C, the DNA pellet was air-dried for 5 min at room temperature, resuspended in 50 µL of nuclease-free water and stored at − 30 °C.

Total RNA was purified from bacterial pellets resuspended in 1 mL of TRIzol reagent stored at − 80 °C, according to the manufacturer instructions. RNA was resuspended in nuclease-free water, treated with the Turbo™ DNase (Invitrogen) according to the manufacturer instructions, and stored at − 80 °C.

### Quantification of ROD21 excision and gene expression

The excision of ROD21, expressed as the relative frequency of chromosomes containing an empty *attB* integration site, was measured by quantitative PCR (qPCR) using hydrolysis probes (Supplemental Table S1) and the TaqMan® Fast Advanced Master Mix (Applied Biosystems). The relative frequency resulted from calculating the number of *attB* sites per 100 *rpoD* copies in the sample, and was expressed as percentage. As *rpoD*, which encodes the σ^70^ factor of the RNA polymerase, is a single-copy gene, its quantification approximates the total number of chromosomes. Therefore, the frequency of excision can vary from 0 (all chromosomes have an integrated ROD21) to 100% (all chromosomes have an empty *attB* site). The absolute numbers of both *attB* and *rpoD* in each sample were determined using a standard curve made with ten-fold dilutions of the *Salmonella* ser. Typhimurium 14028s genomic DNA, which lacks ROD21 and contain only one copy of *attB* and *rpoD* per chromosome. The attB-1 probe and primers attB1-RT-Fw/Rev were used for detection of the *attB* site and the rpoD probe and primers rpoD-RT-Fw/Rev for *rpoD*.

Gene expression was quantified by qRT-PCR with hydrolysis probes SEN1970-RT and SEN1998-RT, and the primers SEN1970-RT-Fw/Rev and SEN1998-RT-Fw/Rev (Supplemental Table S1). cDNA was synthesized using the iScript™ cDNA Synthesis Kit (Bio-Rad) according to the manufacturer instructions. Relative expression was calculated by the 2^−(ΔΔCt)^ method using the expression of *rpoD* as the endogenous control, and the wild-type strain grown at 37 °C, at the time of sampling or at time zero, as the calibrator sample. Then, the relative gene expression was plotted as the log_2_(fold change; FC) = log_2_[2^−(ΔΔCt)^].

### Statistical analyses

The differences in the excision of ROD21 and the expression of *SEN1970* between the wild-type, mutant and complemented strains at different concentrations of IPTG, were analyzed by two-way ANOVA followed by the Tukey’s post-hoc test. The correlation between the excision and *SEN1970* expression was assessed by the Pearson’s correlation test. Both analyses were carried out in GraphPad Prism with α = 0.05.

## Supplementary Information

Supplementary Information.

## References

[1] Williams KP (2002). Integration sites for genetic elements in prokaryotic tRNA and tmRNA genes: Sublocation preference of integrase subfamilies. Nucleic Acids Res..

[2] Marcoleta AE, Berríos-Pastén C, Nuñez G, Monasterio O, Lagos R (2016). *Klebsiella pneumoniae* asparagine tDNAs are integration hotspots for different genomic islands encoding microcin E492 production determinants and other putative virulence factors present in hypervirulent strains. Front. Microbiol..

[3] Che D, Hasan MS, Chen B (2014). Identifying Pathogenicity Islands in Bacterial Pathogenomics Using Computational Approaches. Pathogens.

[4] Piña-Iturbe A (2020). Horizontally acquired homologs of xenogeneic silencers: Modulators of gene expression encoded by plasmids phages and genomic islands. Genes.

[5] Regmi A, Boyd EF (2019). Carbohydrate metabolic systems present on genomic islands are lost and gained in *Vibrio parahaemolyticus*. BMC Microbiol..

[6] de Curraize C, Siebor E, Varin V, Neuwirth C, Hall RM (2020). Two New SGI1-LK variants found in *Proteus mirabilis* and evolution of the SGI1-HKL group of *Salmonella* genomic islands. mSphere.

[7] Piña-Iturbe A (2018). Comparative and phylogenetic analysis of a novel family of *Enterobacteriaceae*-associated genomic islands that share a conserved excision/integration module. Sci. Rep..

[8] Salazar-Echegarai FJ, Tobar HE, Nieto PA, Riedel CA, Bueno SM (2014). Conjugal transfer of the pathogenicity island ROD21 in *Salmonella enterica* serovar Enteritidis depends on environmental conditions. PLoS ONE.

[9] Fillol-Salom A (2019). Hijacking the hijackers: *Escherichia coli *Pathogenicity Islands redirect helper phage packaging for their own benefit. Mol. Cell.

[10] Neale HC, Jackson RW, Preston GM, Arnold DL (2018). Supercoiling of an excised genomic island represses effector gene expression to prevent activation of host resistance. Mol. Microbiol..

[11] Tobar HE (2013). Chromosomal excision of a new pathogenicity island modulates *Salmonella* virulence *in vivo*. Curr. Gene Ther..

[12] Landy A (2015). The λ integrase site-specific recombination pathway. Microbiol. Spectr..

[13] Seah NE (2014). Nucleoprotein architectures regulating the directionality of viral integration and excision. Proc. Natl. Acad. Sci. U. S. A..

[14] Laxmikanthan G (2016). Structure of a holliday junction complex reveals mechanisms governing a highly regulated DNA transaction. Elife.

[15] Guarneros G, Echols H (1970). New mutants of bacteriophage λ with a specific defect in excision from the host chromosome. J. Mol. Biol..

[16] Moitoso de Vargas L, Landy A (1991). A switch in the formation of alternative DNA loops modulates λ site-specific recombination. Proc. Natl. Acad. Sci. U. S. A..

[17] Daccord A, Mursell M, Poulin-laprade D, Burrus V (2012). Dynamics of the SetCD-regulated integration and excision of genomic islands mobilized by integrating conjugative elements of the SXT/R391 family. J. Bacteriol..

[18] Poulin-Laprade D, Matteau D, Jacques PÉ, Rodrigue S, Burrus V (2015). Transfer activation of SXT/R391 integrative and conjugative elements: Unraveling the SetCD regulon. Nucleic Acids Res..

[19] Daccord A, Ceccarelli D, Burrus V (2010). Integrating conjugative elements of the SXT/R391 family trigger the excision and drive the mobilization of a new class of *Vibrio* genomic islands. Mol. Microbiol..

[20] Daccord A, Ceccarelli D, Rodrigue S, Burrus V (2013). Comparative analysis of mobilizable Genomic Islands. J. Bacteriol..

[21] Kiss J (2015). The master regulator of IncA/C plasmids is recognized by the *Salmonella* Genomic Island SGI1 as a signal for excision and conjugal transfer. Nucleic Acids Res..

[22] Haskett TL (2016). Assembly and transfer of tripartite integrative and conjugative genetic elements. Proc. Natl. Acad. Sci. U. S. A..

[23] Haskett TL (2018). Sequential induction of three recombination directionality factors directs assembly of tripartite integrative and conjugative elements. PLOS Genet..

[24] Thomson NR (2008). Comparative genome analysis of *Salmonella* Enteritidis PT4 and *Salmonella* Gallinarum 287/91 provides insights into evolutionary and host adaptation pathways. Genome Res..

[25] Quiroz TS (2011). Excision of an unstable pathogenicity island in *Salmonella enterica* serovar Enteritidis is induced during infection of phagocytic cells. PLoS ONE.

[26] Newman RM, Salunkhe P, Godzik A, Reed JC (2006). Identification and characterization of a novel bacterial virulence factor that shares homology with mammalian toll/interleukin-1 receptor family proteins. Infect. Immun..

[27] Xiong D (2019). *Salmonella* coiled-coil- and TIR-containing TcpS evades the innate immune system and subdues inflammation. Cell Rep..

[28] Godfrey SAC (2011). The Stealth Episome: Suppression of gene expression on the excised genomic island PPHGI-1 from *Pseudomonas syringae* pv. *phaseolicola*. PLoS Pathog..

[29] Nieto PA (2016). New insights about excisable pathogenicity islands in *Salmonella* and their contribution to virulence. Microbes Infect..

[30] Adeolu M, Alnajar S, Naushad S, Gupta RS (2016). Genome-based phylogeny and taxonomy of the ‘Enterobacteriales’: Proposal for Enterobacterales ord. nov. divided into the families *Enterobacteriaceae*, *Erwiniaceae* fam. nov., *Pectobacteriaceae* fam. nov., *Yersiniaceae* fam. nov., *Hafniaceae* fam. nov., *Morganellaceae *fam. nov., and* Budviciaceae* fam. nov. Int. J. Syst. Evol. Microbiol..

[31] Panis G, Franche N, Méjean V, Ansaldi M (2012). Insights into the functions of a prophage recombination directionality factor. Viruses.

[32] Lewis JA, Hatfull GF (2001). Control of directionality in integrase-mediated recombination: Examination of recombination directionality factors (RDFs) including Xis and Cox proteins. Nucleic Acids Res..

[33] Carpenter MR, Rozovsky S, Boyd EF (2016). Pathogenicity island cross talk mediated by recombination directionality factors facilitates excision from the chromosome. J. Bacteriol..

[34] Sam MD, Cascio D, Johnson RC, Clubb RT (2004). Crystal structure of the excisionase-DNA complex from bacteriophage Lambda. J. Mol. Biol..

[35] ElAntak L, Ansaldi M, Guerlesquin F, Méjean V, Morelli X (2005). Structural and genetic analyses reveal a key role in prophage excision for the TorI response regulator inhibitor. J. Biol. Chem..

[36] Panis G, Méjean V, Ansaldi M (2007). Control and regulation of KplE1 prophage site-specific recombination: A new recombination module analyzed. J. Biol. Chem..

[37] Abbani MA (2007). Structure of the cooperative Xis-DNA complex reveals a micronucleoprotein filament that regulates phage lambda intasome assembly. Proc. Natl. Acad. Sci. USA.

[38] Ramsay JP, Sullivan JT, Stuart GS, Lamont IL, Ronson CW (2006). Excision and transfer of the *Mesorhizobium loti* R7A symbiosis island requires an integrase IntS, a novel recombination directionality factor RdfS, and a putative relaxase RlxS. Mol. Microbiol..

[39] Carraro N (2011). Differential regulation of two closely related integrative and conjugative elements from *Streptococcus thermophilus*. BMC Microbiol..

[40] Lautner M, Schunder E, Herrmann V, Heuner K (2013). Regulation, integrase-dependent excision, and horizontal transfer of genomic islands in *Legionella pneumophila*. J. Bacteriol..

[41] Lesic B (2004). Excision of the high-pathogenicity island of *Yersinia pseudotuberculosis* requires the combined of actions of its cognate integrase and Hef, a new recombination directionality factor. Mol. Microbiol..

[42] Antonenka U, Nölting C, Heesemann J, Rakin A (2006). Independent acquisition of site-specific recombination factors by *asn* tRNA gene-targeting genomic islands. Int. J. Med. Microbiol..

[43] Panis G (2010). Tight regulation of the *intS* gene of the KplE1 prophage: A new paradigm for integrase gene regulation. PLoS Genet..

[44] Bowers JR (2015). Genomic analysis of the emergence and rapid global dissemination of the clonal group 258 *Klebsiella pneumoniae* pandemic. PLoS ONE.

[45] Zhang X (2019). New Delhi metallo-β-lactamase 5–producing *Klebsiella pneumoniae* sequence type 258, Southwest China, 2017. Emerg. Infect. Dis..

[46] Chen L, Mathema B, Pitout JDD, DeLeo FR, Kreiswirth BN (2014). Epidemic *Klebsiella pneumoniae* ST258 is a hybrid strain. mBio.

[47] Carraro N, Rivard N, Ceccarelli D, Colwell RR, Burrus V (2016). IncA/C conjugative plasmids mobilize a new family of multidrug resistance islands in clinical *Vibrio cholerae* non-O1/non-O139 isolates from Haiti. mBio.

[48] McKitterick AC, Seed KD (2018). Anti-phage islands force their target phage to directly mediate island excision and spread. Nat. Commun..

[49] Penadés JR, Christie GE (2015). The phage-inducible chromosomal islands: A family of highly evolved molecular parasites. Annu. Rev. Virol..

[50] Datsenko KA, Wanner BL (2000). One-step inactivation of chromosomal genes in *Escherichia coli* K-12 using PCR products. Proc. Natl. Acad. Sci. U. S. A..

[51] Cherepanov PP, Wackernagel W (1995). Gene disruption in *Escherichia coli*: Tc^R^ and Km^R^ cassettes with the option of Flp-catalyzed excision of the antibiotic-resistance determinant. Gene.

[52] Kumar S, Stecher G, Li M, Knyaz C, Tamura K (2018). MEGA X: Molecular evolutionary genetics analysis across computing platforms. Mol. Biol. Evol..

[53] Waterhouse AM, Procter JB, Martin DMA, Clamp M, Barton GJ (2009). Jalview Version 2-A multiple sequence alignment editor and analysis workbench. Bioinformatics.

[54] Roy A, Kucukural A, Zhang Y (2010). I-TASSER: A unified platform for automated protein structure and function prediction. Nat. Protoc..

[55] Madeira F (2019). The EMBL-EBI search and sequence analysis tools APIs in 2019. Nucleic Acids Res..

[56] Trifinopoulos J, Nguyen L, von Haeseler A, Minh BQ (2016). W-IQ-TREE: A fast online phylogenetic tool for maximum likelihood analysis. Nucleic Acids Res..

[57] Minh BQ, Nguyen MAT, von Haeseler A (2013). Ultrafast approximation for phylogenetic bootstrap. Mol. Biol. Evol..

[58] Schneider CA, Rasband WS, Eliceiri KW (2012). NIH Image to ImageJ: 25 years of image analysis. Nat. Methods.

