# Supplementary material for: Bioinformatic and experimental characterization of SEN1998: a conserved gene carried by the Enterobacteriaceae-associated ROD21-like family of genomic islands
Source: Sci Rep. 2022 Feb 14;12:2435. doi: 10.1038/s41598-022-06183-x (PMC8844411; doi:10.1038/s41598-022-06183-x)
Supplement: Supplementary file 1 — Supplementary Information. [file 41598_2022_6183_MOESM1_ESM.pdf]

**Supplemental Material**

# Bioinformatic and experimental characterization of *SEN1998*: a conserved gene carried by the *Enterobacteriaceae*-associated ROD21-like family of genomic islands

Alejandro Piña-Iturbe[a], Guillermo Hoppe-Elsholz[a], Paulina A. Fernández[b], Carlos A. Santiviago[b], Pablo A. González[a] and Susan M. Bueno[a]*

[a] Millennium Institute on Immunology and Immunotherapy, Facultad de Ciencias Biológicas, Departamento de Genética Molecular y Microbiología, Pontificia Universidad Católica de Chile, Santiago, Chile

[b] Laboratorio de Microbiología, Departamento de Bioquímica y Biología Molecular, Facultad de Ciencias Químicas y Farmacéuticas, Universidad de Chile, Santiago, Chile.

*Address correspondence to Susan M. Bueno, sbueno@bio.puc.cl

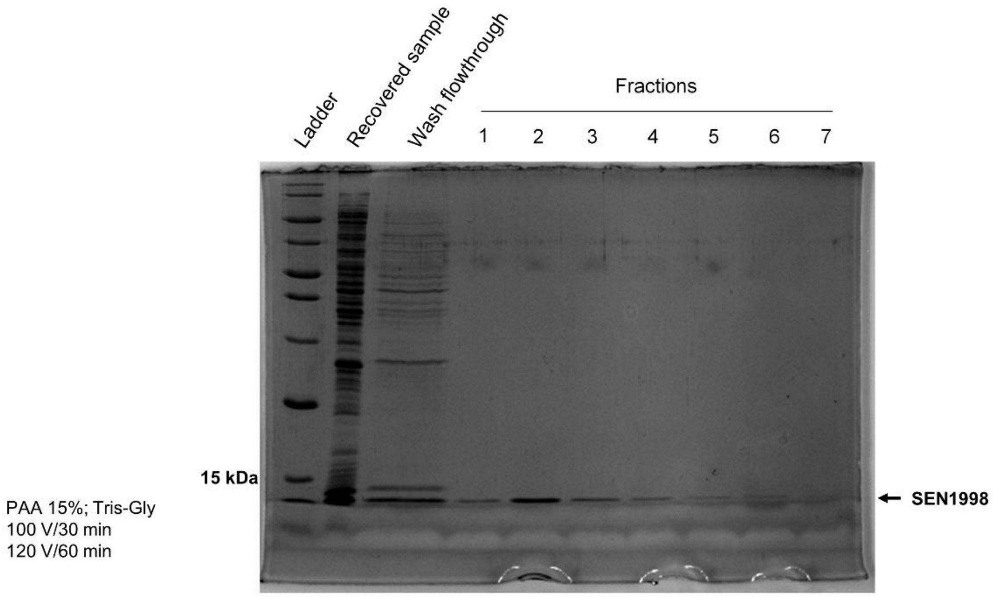


**Figure S1. IMAC purification of the SEN1998 protein.** Coomassie-stained polyacrylamide gel loaded with samples and fractions recovered after IMAC. Lane 1: molecular-weight marker; lane 2: *Escherichia coli* BL21(DE3) pET15b-*SEN1998* lysate after IMAC; lane 3: recovered wash flowthrough; lane 4-10: fractions eluted from the column.

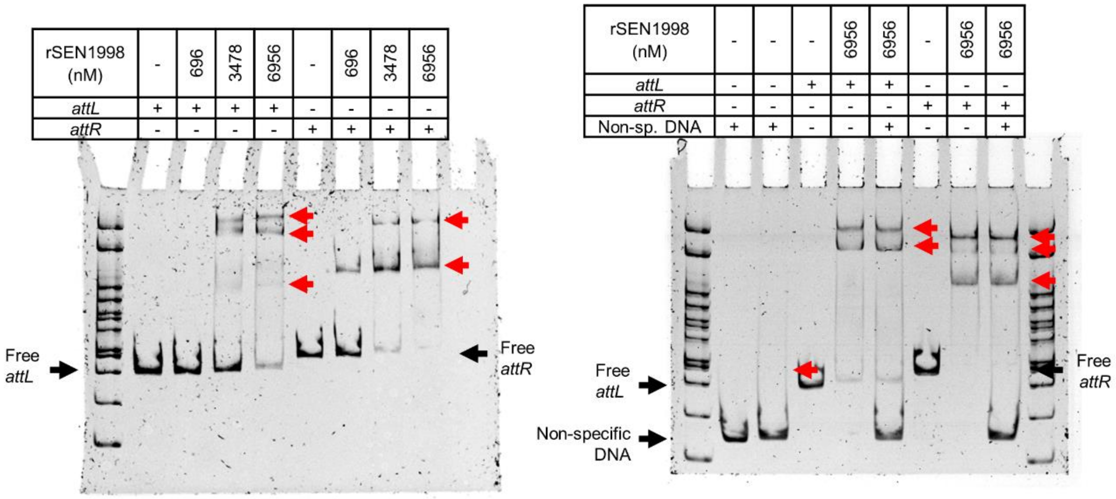


**Figure S2. Full length gels for figure 4.** Not cropped versions of the polyacrylamide gels showing the EMSA results of Figure 4. The black and red arrows indicate the free and complexed target DNA.

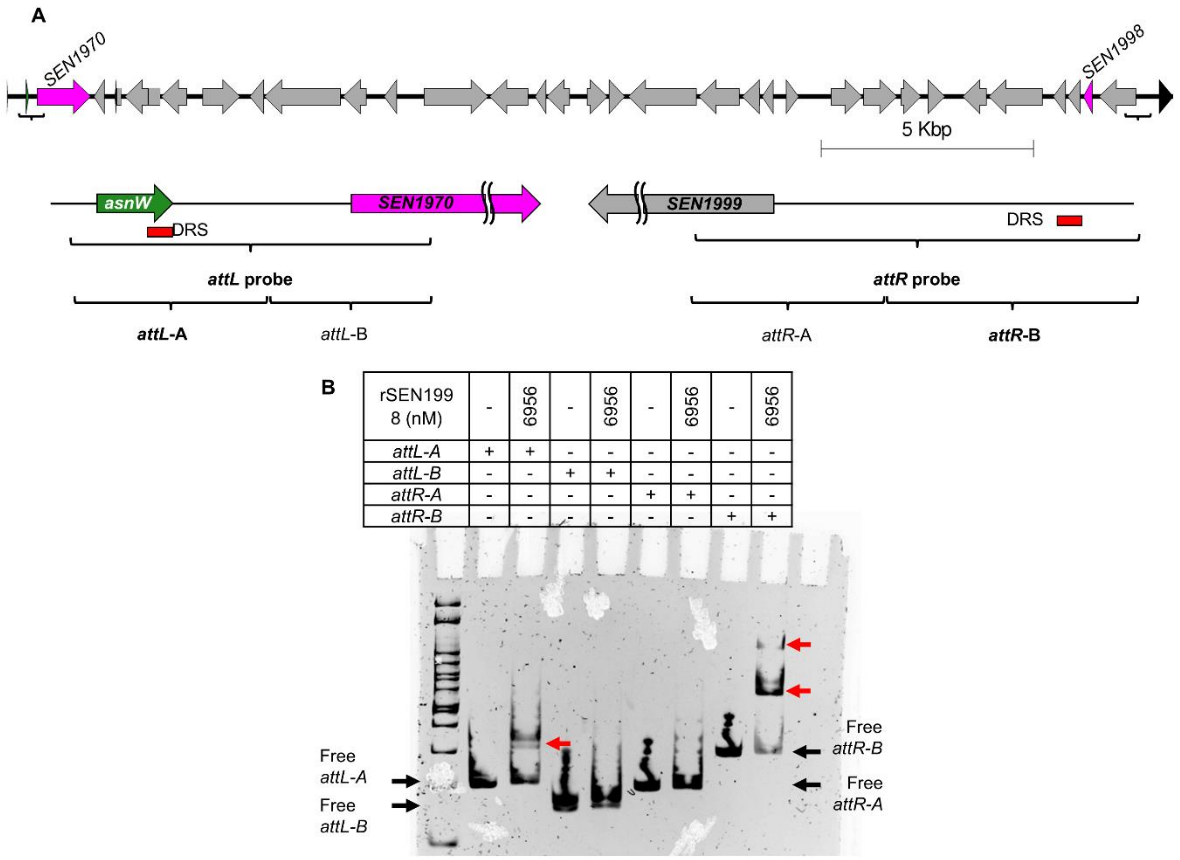


**Figure S3. SEN1998 bins the most distal regions of *attL* and *attR*.** (**A**) Schematic representation of the target DNAs encompassing the left and right attachment regions of ROD21 used for the EMSA. (**B**) EMSA showing the binding of SEN1998 to the *attL*-A and *attR*-B subregions. The black and red arrows indicate the free and complexed target DNA.

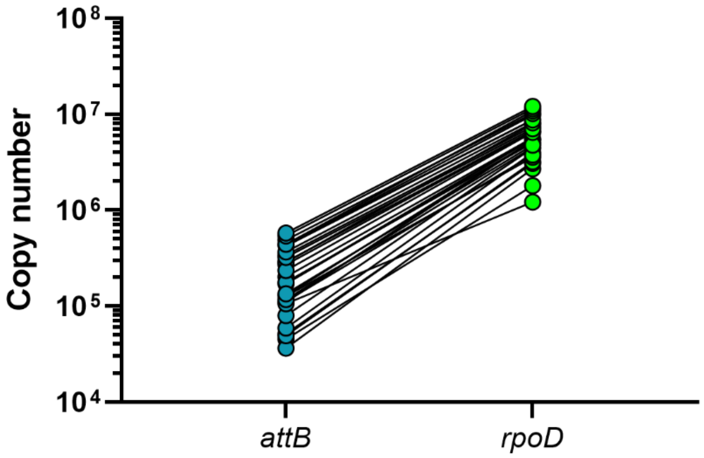


**Figure S4. Correspondence between the *attB* and *rpoD* copy numbers.** The attB and rpoD copies estimated by qPCR and used to calculate the excision of ROD21 in Figure 5 were plotted. Copy-numbers of *attB* and *rpoD* from the same sample are connected by a line. It can be noted that the higher the *rpoD* copy-number, the higher the *attB* copy-number in for the same sample.

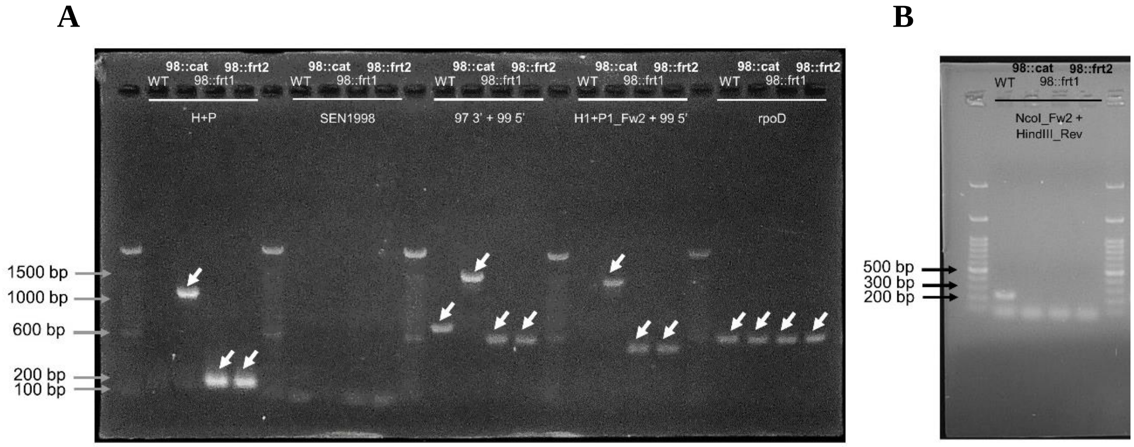


| Primers | WT | *Δ98:cat* | *Δ98:FRT* |
|---|---|---|---|
| H+P | -- | 1094 bp | 164 bp |
| SEN1998 | 235 bp | -- | -- |
| 97 3'+ 99 5' | 702 bp | 1497 bp | 567 bp |
| H1P1+99 5' | -- | 1406 bp | 476 bp |
| rpoD | 577 bp | 577 bp | 577 bp |

**Figure S5. PCR confirmation of the Δ*SEN1998*::*frt* deletion.** (**A**) Deletion of the *SEN1998* coding sequence was assessed by PCR using specific and external primers (see also Table S1). (**B**) The PCR using primers specific for *SEN1998* was repeated with a new set of primers since no amplification of the positive control was observed in the previous PCR. Bands were obtained at the expected size, confirming the deletion of *SEN1998*.

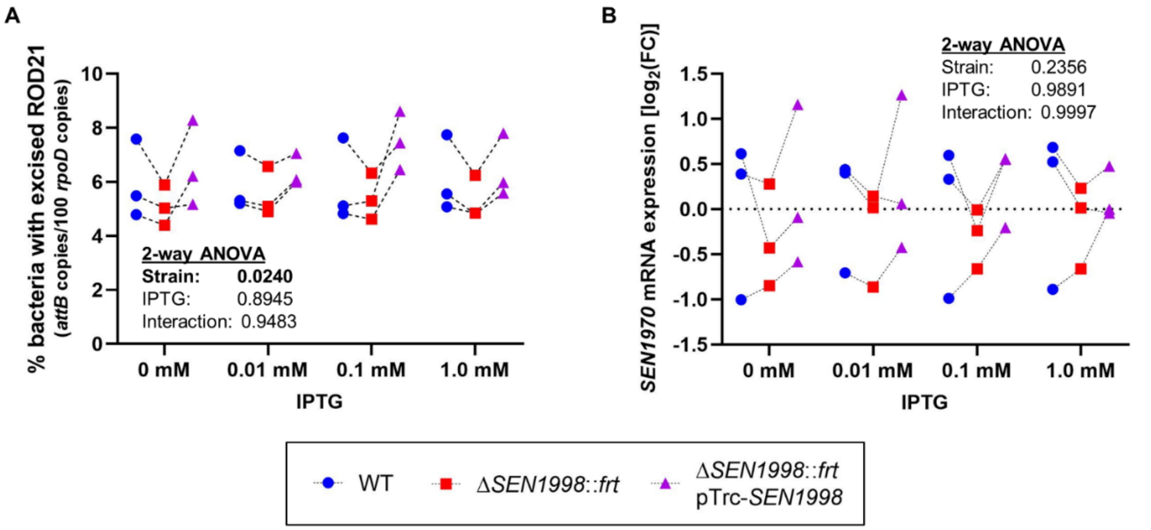


**Figure S6. Excision of ROD21 and expression of *SEN1970* in the *Salmonella* ser. Enteritidis wild-type, Δ*SEN1998*::*frt* and Δ*SEN1998*::*frt* pTrc-SEN1998 strains.** (**A**) Excision levels of ROD21. (**B**) mRNA levels of the integrase-encoding gene *SEN1970* in the three strains, relative to the levels in the P125109 wild-type strain. This is the same figure as Fig. 5A-B, but the bars were omitted and the individual excision or expression trends are depicted by broken lines.

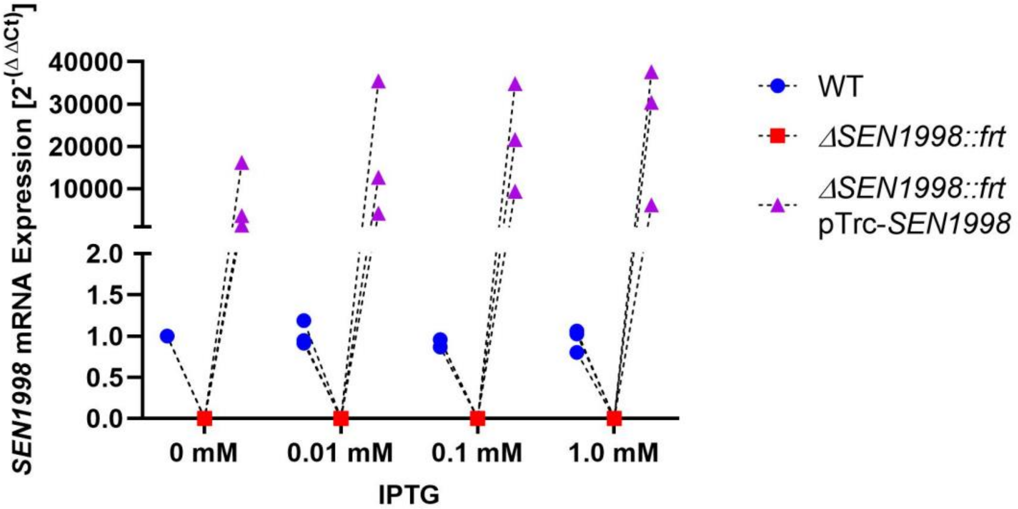


**Figure S7. Expression of *SEN1998* in the wildtype, Δ*SEN1998*::*frt*, and Δ*SEN1998*::*frt* pTrc-*SEN1998* strains**. The three bacterial strains were grown in the absence or presence of IPTG, used as the inducer for the expression of *SEN1998* from plasmid pTrc-SEN1998. The mRNA levels of *SEN1998*, relative to the wild-type strain were measured by RT-qPCR. Each symbol corresponds to one independent experiment.

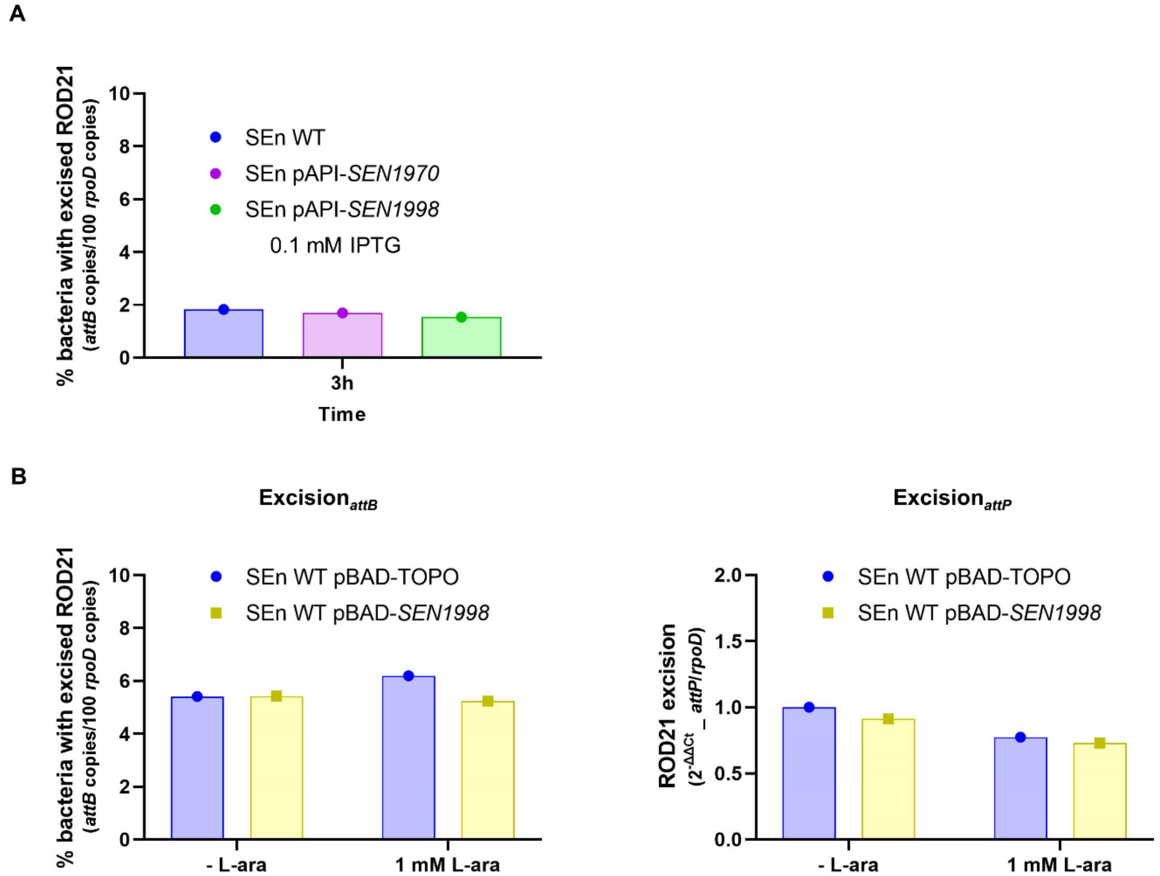


**Figure S8. Excision of ROD21 in the *Salmonella* ser. Enteritidis wild-type, pAPI-*SEN1998* and pBAD-*SEN1998* strains.** The effect of the *SEN1998* expression from an (**A**) IPTG- and (**B**) L-arabinose-inducible promoters were assessed in the *Salmonella* ser. Enteritidis wild-type strain transformed with the corresponding plasmids. 0.1 mM IPTG and 1 mM L-arabinose were used to induce the expression in 5 mL cultures. For the pBAD-TOPO (empty vector) and pBAD-*SEN1998* strains, the excision was estimated as the number of *attB* copies/100 *rpoD* copies (**B; Excision$_{attB}$, left panel**) and as the quantification of the *attP* copies relative to the *rpoD* copies, normalized to the uninduced empty-vector strain ($2^{-\Delta\Delta Ct}$) (**B; Excision$_{attP}$, right panel**). The Excision$_{attP}$ was calculated this way because a standard curve for absolute quantification is not available.

**Table S1. Sequences of the primers and hydrolysis probes used in this study.**

| **Primers** | **Sequence (5'->3')** | **Use** |
|---|---|---|
| SEN1998 _H1+P1_Fw2 | CGA ACA GAT ATC TGT TCG ACA CGA GAT CCT ATT TCT GTT Tgt gta ggc tgg agc tgc ttc | Construction of mutant strain Δ*SEN1998*::*frt* and confirmation |
| SEN1998_H2+P2_Rev2 | TCC ATA CAA TTA TCT CCA TCG AAT AAC ACA GGA GAT AAA Tca tat gaa tat cct cct tag | Construction of mutant strain Δ*SEN1998*::*frt* and confirmation |
| SEN1998_NcoI_Fw2 | GCA GCC ATG GTT AAA AGA GAA ATA AAA GC | Amplification of *SEN1998* |
| SEN1998_HindIII_Rev | GTT TAA GCT TTT ATG CTG CGT TCT GGC | Amplification of *SEN1998* |
| 3' 1997 Fw | CAT TCG GCT TGT TCA ATA AA | Amplification of the region containing *SEN1998* |
| 5' 1999 Rev | TAT GCG TTA TGC CAG AGG TA | Amplification of the region containing *SEN1998* |
| SEN1970_H1+P1_Fw2 | GGT TCG ATT TGT GTT TTA CCA GCA CGC GGA GGG AAC CGT Cgt gta ggc tgg agc tgc ttc | Construction of mutant strain Δ*SEN1970*::*frt* and confirmation |
| SEN1970_H2+P2_Rev | TTT TTT GAT TTA ATG CGA TTG TTT GCT TGT AGG AAG GTG Aca tat gaa tat cct cct tag | Construction of mutant strain Δ*SEN1970*::*frt* and confirmation |
| SEN1970_NdeI_Fw | TTG ACG CAT ATG TCA CTT ACT GAT ACC AAA G | Amplification of *SEN1970* |
| SEN1970_BamHI_Rev | TAT TCG GGA TCC TTA TTG TTT CTG AGC AAA C | Amplification of *SEN1970* |
| attR_Rev | TTA AAC GTC TCC GGA CTC GCC | Amplification of target DNA for EMSA |
| attR-A_Rev | ACA GAT TAC CGC TAT TGC CCG | Amplification of target DNA for EMSA |
| attR-B_Fw | GAA ACA TAT CGG GCA ATA GCG G | Amplification of target DNA for EMSA |
| attR_Fw | CGC GAC AGG CAA TCT TTT TGT C | Amplification of target DNA for EMSA |
| attL_Fw | TAC TAT GCG CCC CGT TCA CAC | Amplification of target DNA for EMSA and confirmation of Δ*SEN1970*::*frt* strain. |
| attL_Rev | AT AGA GGC CAA ACC CGT CAG TG | Amplification of target DNA for EMSA |
| attL-B_Fw | GA AGC CAT ACC CTT TTG CTG G | Amplification of target DNA for EMSA |
| attL-A_Rev | GA GTG GTT TTT AGT GAA CGA GAG TG | Amplification of target DNA for EMSA |
| SEN1998-RT_Fw | GAA AAC GCC CGG CCT TAA | Quantification of the *SEN1998* mRNA expression |
| SEN1998-RT_Rev | CGC ACC GGA TTT GGT AAA A | Quantification of the *SEN1998* mRNA expression |
| SEN1970-RT_Fw | CGA TAC TGT CTG GAA GCG CCT | Quantification of the *SEN1970* mRNA expression |
| SEN1970-RT_Rev | TTT TGC TGG ACG GCA TGA C | Quantification of the *SEN1970* mRNA expression |
| rpoD-RT_Fw | GTT GAC CCG GGA AGG CGA AA | Quantification of the ROD21 excision |
| rpoD-RT_Rev | CAG AAC CGA CGT GAG TTG CG | Quantification of the ROD21 excision |
| attB1-RT_Fw | GTT ACT ATG CGC CCC GTT CAC AC | Quantification of the ROD21 excision |
| attB1-RT_Rev | CCG ATT AAG CCC CAA AAA CTA TG | Quantification of the ROD21 excision |
| **Probes** | **Sequence (5'->3')** | **Use** |
| attB1-RT | TTC GAG TCC AGT CAG AGG A | Quantification of the ROD21 excision |
| rpoD-RT | CGA CAT CGC TAA ACG | Quantification of the ROD21 excision |
| SEN1970-RT | TCA CCG CGA TCC TA | Quantification of the *SEN1970* mRNA expression |
| SEN1998-RT | CAG TTC GTA AAT CCA C | Quantification of the *SEN1998* mRNA expression |
